# *Phellinus igniarius* Polysaccharides Ameliorate Hyperglycemia by Modulating the Composition of the Gut Microbiota and Their Metabolites in Diabetic Mice

**DOI:** 10.3390/molecules28207136

**Published:** 2023-10-17

**Authors:** Zaizhong Ni, Jinting Li, Xinyi Qian, Yidan Yong, Mengmeng Wu, Yanan Wang, Wendi Lv, Simeng Zhang, Yifei Zhang, Ying Shao, Anhui Chen

**Affiliations:** 1 College of Food and Bioengineering, Xuzhou University of Technology, Xuzhou 221018, China; 2 College of Pharmacy, Nanjing University of Chinese Medicine, Nanjing 210023, China

**Keywords:** *Phellinus igniarius*, polysaccharide, hypoglycemia, gut microbiota, metabolites, fetal microbiota transplantation

## Abstract

Gut microbiota dysbiosis has been reported as a risk factor in the development of type 2 diabetes mellitus (T2DM). Polysaccharides from *Phellinus igniarius* (*P. igniarius*) possess various properties that help manage metabolic diseases; however, their underlying mechanism of action remains unclear. Therefore, in this study, we aimed to evaluate the effect of *P. igniarius* polysaccharides (SH-P) on improving hyperglycemia in mice with T2DM and clarified its association with the modulation of gut microbiota and their metabolites using 16S rDNA sequencing and liquid chromatography–mass spectrometry. Fecal microbiota transplantation (FMT) was used to verify the therapeutic effects of microbial remodeling. SH-P supplementation alleviated hyperglycemia symptoms in T2DM mice, ameliorated gut dysbiosis, and significantly increased the abundance of *Lactobacillus* in the gut. Pathway enrichment analysis indicated that SH-P treatment altered metabolic pathways associated with the occurrence and development of diabetes. Spearman’s correlation analysis revealed that changes in the dominant bacterial genera were significantly correlated with metabolite levels closely associated with hyperglycemia. Additionally, FMT significantly improved insulin sensitivity and antioxidative capacity and reduced inflammation and tissue injuries, indicating improved glucose homeostasis. These results indicate that the ameliorative effects of SH-P on hyperglycemia are associated with the modulation of gut microbiota composition and its metabolites.

## 1. Introduction

Type 2 diabetes mellitus (T2DM), which accounts for 90–95% of all diabetes, is a chronic metabolic disease characterized by insulin resistance and relative insulin insufficiency and has become one of the most prevalent public health challenges worldwide [1,2]. Different types of oral hypoglycemic agents, including insulin, thiazolidinediones, sulfonylureas, glucagon-like peptide-1 (GLP-1) agonists, dipeptidyl peptidase-4 inhibitors, metformin, meglitinides, sodium-glucose cotransporter 2 inhibitors, and pramlintide are used to treat T2DM [3]. However, the targets and pathways associated with these drugs are relatively simple and long-term use can produce many adverse effects. Therefore, developing safer and effective drugs for the treatment of T2DM is necessary.

Natural compounds have become a new prevention and treatment approach for T2DM and can attenuate the complications of hyperglycemia owing to their non-toxicity, cost-effectiveness, and easy absorption [4,5]. *P. igniarius* is a rare, large, perennial, edible, and medicinal fungus that belongs to *Basidiomycotina*, *Hymenomycetes*, *Aphyllophorales*, and *Hymenochaetaceae* and is commonly referred to as “forest gold” [4,6]. According to the literature, *P. igniarius* is used to treat diarrhea, promote blood circulation, improve gastroenteric dysfunction, and treat cancer [7]. *P. igniarius* contains various bioactive substances, including polysaccharides, flavonoids, styrylpyrones, and phenolic compounds [8]. Emerging evidence has demonstrated that polysaccharides isolated from *P. igniarius* exhibit significant hypoglycemic activity, as oral administration of these polysaccharides lowered blood glucose levels and stimulated insulin excretion in streptozocin (STZ)-induced diabetic rats, consequently restoring pancreas, liver, and kidney functions [9]. These polysaccharides also exhibit a considerable hypoglycemic effect and improve insulin sensitivity, possibly through regulating peroxisome proliferator-activated receptor (PPAR)-γ-mediated lipid metabolism [10]. However, the mechanism by which these polysaccharides improve hyperglycemia symptoms requires further exploration.

Emerging evidence indicates that disturbances in the gut microbiota are associated with the development of diabetes and related metabolic disorders [11]. For example, butyrate-producing bacteria are negatively correlated with hyperglycemic parameters, *Lactobacilli* are more prevalent in T2DM patients, and the class Clostridia and phylum Firmicutes are significantly positively correlated with inflammation [12]. A metabolite-based genome-wide association study analysis showed that the gut microbiota was dysbiotic in patients with T2DM, the abundance of many butyrate-producing bacteria decreased, and the abundance of various opportunistic pathogens increased [13]. Gram-negative bacteria, which are relatively enriched in the gut, are closely related to low-grade inflammation and insulin resistance in patients with T2DM. *Prevotella copri* and *Bacteroides vulgatus* can be used as characteristic bacteria to predict the occurrence and development of diabetes [14]. Therefore, gut microbiota may serve as a potential new target for diabetes therapy. However, it is still unclear whether pathogenic bacteria result in the occurrence of T2DM or the changes in gut microbiota caused by T2DM lead to an increase in pathogenic bacteria, promoting the development of T2DM. Therefore, analyzing the characteristics of the gut microbiota in patients with T2DM and elucidating the microbiome changes of T2DM before and after treatment will help delineate the role of gut bacteria in the development of T2DM.

Fecal microbiota transplantation (FMT) is an effective method for reestablishing the gut microbiota by transplanting the intestinal microbial community of a healthy donor into the gastrointestinal tract of the recipient, thereby rapidly changing the recipient’s gut microbiota composition [15]. An oral glucose tolerance test (OGTT) showed that FMT significantly decreased blood glucose and glycated hemoglobin (HbA1c) levels, improved insulin resistance, and increased insulin sensitivity in T2DM mice [16]. FMT treatment also significantly decreased the kidney to body weight ratios, urinary albumin to creatinine, and urinary N-acetyl-β glucosaminase to creatinine and relieved desquamation and necrosis of renal tubular epithelial cells in diabetic rats [17]. In addition, transplantation of the intestinal microbiota of normal rats into obese diabetic rats significantly decreases body weight and improves insulin and leptin resistance via the Janus kinase 2 (JAK2)/insulin receptor substrate (IRS)/protein kinase B (Akt) pathway [18]. However, the specific pathophysiological role of the microbial community and its underlying mechanism of action, and the efficacy of fecal or intestinal microbiota trans-plantation as a method to treat T2DM remains unclear. Elucidating these processes will provide a theoretical basis for further optimizing the feasibility of microbiota transplantation methods and developing appropriate microbiota regulation techniques.

Recent studies have demonstrated that active polysaccharides can improve T2DM by regulating the composition of intestinal microbiota and the production of bacteria-derived metabolites [19]. *Cordyceps militaris* polysaccharides alleviate diabetic symptoms by regulating gut microbiota against the toll-like receptor 4 (TLR4)/nuclear factor kappa B (NF-κB) pathway [20]. Polysaccharides extracted from small black soybeans alleviate T2DM by modulating the gut microbiota and serum metabolism [21]. *Brasenia schreberi* polysaccharides improve hyperglycemia symptoms and reverse gut microbiota dysbiosis by enhancing the abundance of *Lactobacillus* to activate phosphatidylinositol 3-kinase (PI3K)/Akt-mediated signaling pathways in T2DM mice [22]. Although polysaccharides isolated from *P. igniarius* have hypoglycemic functions, their effects on the gut microbiota and their metabolites remain unclear.

In this study, the hypoglycemic effect of polysaccharides isolated from *P. igniarius* and their regulatory effects on the structure and characteristics of the gut microbiota and their metabolites in high-fat diet/STZ-induced diabetic mice was evaluated. In addition, FMT was used to investigate the therapeutic effects of feces from diabetic mice treated with polysaccharides from *P. igniarius*. This study provides a reference for the development of new natural polysaccharide drugs to treat diabetes mellitus and its related complications.

## 2. Results

### 2.1. SH-P Treatment Alleviated Hyperglycemia Symptoms in T2DM Mice

**Figure 1:**
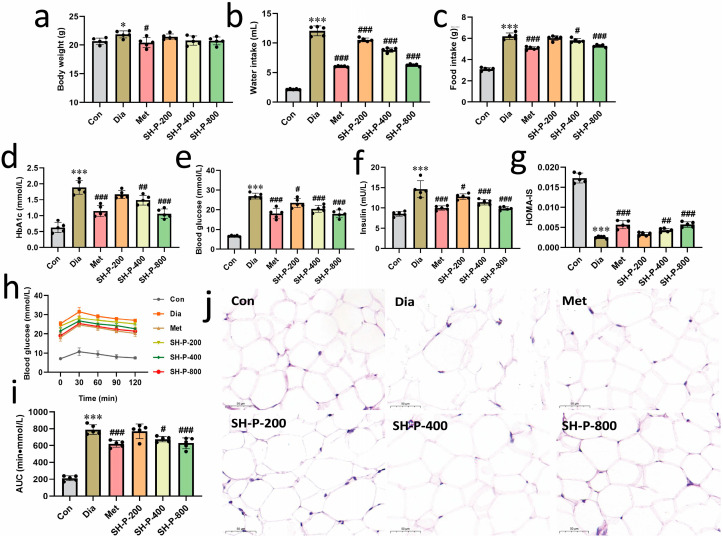
The effects of SH-P treatment on improving hyperglycemia symptoms in type 2 diabetes mellitus (T2DM) mice. (**a**) Body weight; (**b**) Food intake; (**c**) Water intake; (**d**) HbA1c; (**e**) Blood glucose; (**f**) Insulin; (**g**) Homeostasis model assessment of insulin sensitivity (HOMA-IS); (**h**) Oral glucose tolerance test (OGTT); (**i**) Area under the curve (AUC); (**j**) Representative images of H&E-stained adipose tissue. *, *p* < 0.05 vs. Con; ***, *p* < 0.001 vs. Con; #, *p* < 0.05 vs. Dia; ##, *p* < 0.01 vs. Dia; ###, *p* < 0.001 vs. Dia. Scale bars = 50 μm.

The hypoglycemic effects of polysaccharides isolated from *P. igniarius* were evaluated in high-fat diet/STZ-induced diabetic mice. Although no significant difference in body weight was observed, SH-P treatment significantly decreased the food and water intake of diabetic mice in a dose-dependent manner after four weeks of treatment compared to that in the T2DM group (Figure 1a–c). The HbA1c level was significantly lower in SH-P-treated mice, decreasing by 43.80% (*p* < 0.001) compared with the diabetic control mice at 800 mg/kg SH-P (Figure 1d). Moreover, SH-P treatment significantly reduced blood glucose and insulin levels, an effect that was positively correlated with SH-P concentration; these levels decreased by 33.60% (*p* < 0.001) and 32.56% (*p* < 0.001), respectively, at a dosage of 800 mg/kg (Figure 1e,f). Additionally, SH-P treatment significantly improved insulin sensitivity compared with the diabetic control group (0.0057 ± 0.00066 vs. 0.0026 ± 0.00031, *p* < 0.001; Figure 1g). The OGTT indicated that SH-P administration significantly improved glucose tolerance (Figure 1h), and the AUC was reduced by 16.23% compared with that of diabetic mice at 800 mg/kg SH-P (Figure 1i). Moreover, SH-P administration reversed the abnormally enlarged and irregularly arranged adipose cells (Figure 1j).

### 2.2. SH-P Treatment Changed the Microbiota Composition in T2DM Mice

**Figure 2:**
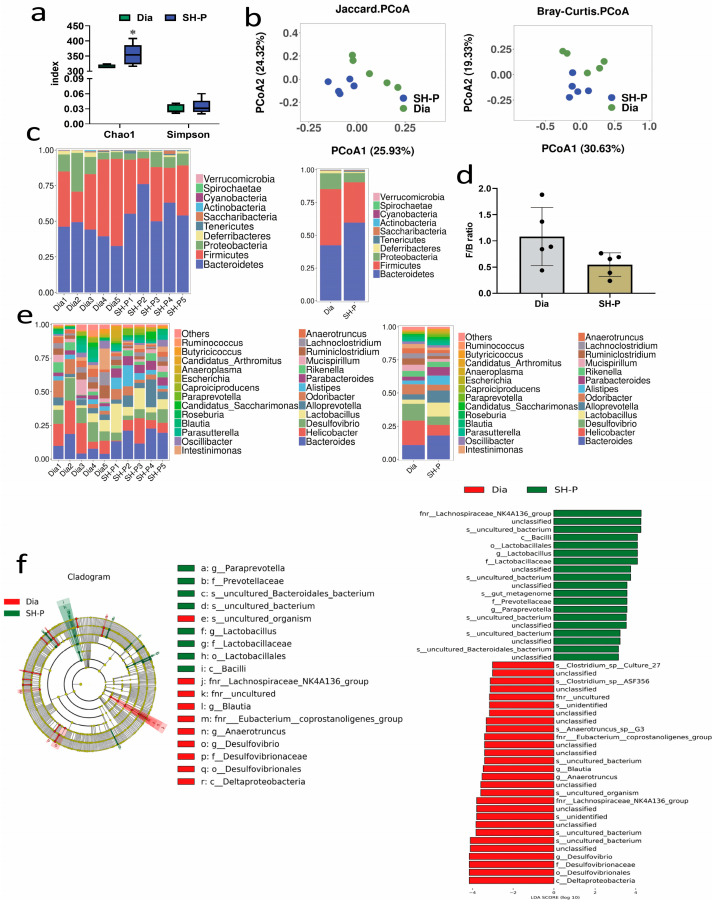
Changes in the diversity and relative abundance in the gut microbiota between diabetic and SH-P-treated mice. (**a**) The alpha diversity of gut microbiota based on Chao1 and Simpson indices; (**b**) Principal coordinates analysis (PCoA) of the gut microbial communities based on Jaccard and Bray–Curtis distances; (**c**) The relative abundance of the gut microbiota at the phylum level; (**d**) Firmicutes/Bacteroidetes (F/B) ratio; (**e**) Relative abundance of the gut microbiota at the genus level; (**f**) Linear discriminant analysis Effect Size (LEfSe) analysis of the taxon at significantly different levels between the gut microbiota of diabetic and SH-P-treated mice. The cladogram showed the microbial species with significant differences (left) and the differences in the abundances of the gut microbiota. The LDA scores were (log10) > 3 and *p* < 0.05 (right). * *p* < 0.05 indicated significant differences.

Next, 16S rDNA sequencing was employed to detect the composition and changes in the gut microbiota of mice following SH-P treatment. The fecal samples used for analyzing the gut microbiota were selected from the SH-P-800 treatment group because SH-P treatment alleviated hyperglycemia symptoms in a dose-dependent manner in T2DM mice. No significant differences were observed in the Simpson index between the diabetic and SH-P-treated groups. The Chao1 index results showed a significant increase after SH-P treatment, indicating an increase in species abundance, richness, and diversity (Figure 2a). The PCoA based on Jaccard and Bray–Curtis distances showed that microbial communities between the diabetic and SH-P-treated groups were clearly separated and clustered into distinct groups, indicating a significant change in gut microbiota composition in response to SH-P treatment (Figure 2b). The gut microbiota was then analyzed at different taxonomic levels. At the phylum level, although the dominant bacterial communities in the fecal samples from both the diabetic and SH-P-treated groups were Bacteroidetes, Firmicutes, Proteobacteria, and Desulfobacterota, the relative proportions of the phyla in the different groups varied considerably (Figure 2c). An increased ratio of Firmicutes/Bacteroidetes (F/B ratio) is a key indicator of microbiota imbalance and can be used to assess the degree of obesity and hyperglycemia [23]; SH-P treatment decreased the F/B ratio compared to the diabetic group, although not significantly (Figure 2d). The relative abundances of other taxonomic levels, such as class, order, family, and species, are shown in Figure S1. At the genus level, compared with the diabetic group, SH-P treatment significantly increased the proportions of *Bacteroides*, *Lactobacillus*, *Alloprevotella*, *Alistipes*, and *Parabacteroides* and significantly decreased the abundances of *Helicobacter*, *Desulfovibrio*, *Odoribacter*, *Mucispirillum*, and *Ruminiclostridium* (Figure 2e).

LEfSe analysis is a comparative method used to investigate specific groups of bacteria that may explain the differences in phenotypes. The LefSe results indicated significant differences at various taxon levels between the diabetic and SH-P-treated groups, including 19 species enriched in the SH-P-treated mice and 28 species enriched in diabetic mice (Figure 2f). The increased abundance was primarily related to an increase in Lactobacillales, *Lactobacillaceae*,_*Paraprevotella*, *Lachnospiraceae NK4A136 group*, *Lactobacillus*, *Prevotellaceae*, Bacilli, uncultured Bacteroidales bacterium, and other unclassified or uncultured bacterium, whereas decreased abundance was mainly associated with Deltaproteobacteria, *Blautia*, *Lachnospiraceae NK4A136 group*, *Eubacterium coprostanoligenes group*, *Anaerotruncus* sp. G3, *Clostridium* sp. ASF356, *Desulfovibrionaceae*, *Anaerotruncus*, *Desulfovibrio*, Desulfovibrionales, and some unclassified or uncultured bacterium in the SH-P-treated mice compared with that in the diabetic mice (Figure 2f). Notably, SH-P treatment significantly increased the abundance of *Lactobacillus* from the phylum to the genus level.

### 2.3. SH-P Treatment Improved the Fecal Metabolomic Profile in T2DM Mice

**Figure 3:**
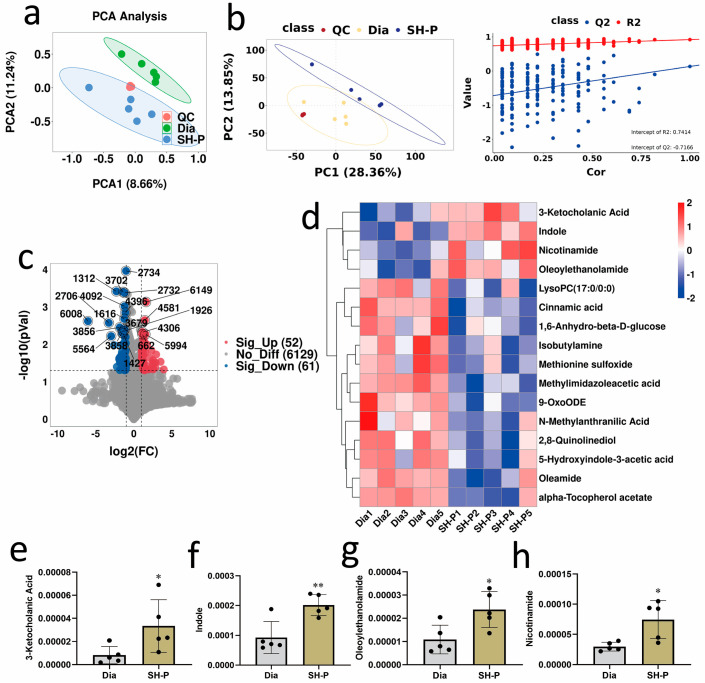
Analysis of differential metabolites by liquid chromatography–mass spectrometry (LC-MS) between diabetic and SH-P-treated mice in ESI-positive (POS) detection mode. (**a**) Score plot of principal component analysis (PCA) showing comparisons of the metabolomic profiles; (**b**) Partial least squares discriminant analysis (PLS-DA) score plot of metabolomic features and validation of the PLS-DA model by permutation testing; (**c**) The deferentially expressed metabolite analysis by volcano plot. The up-regulated metabolites are indicated in red, the down-regulated metabolites are indicated in blue, and the gray dots denote metabolites with insignificant changes in expression; (**d**) A hierarchical clustering heatmap exhibiting the metabolites that significantly differ in abundance; (**e**) Relative abundance of 3-Ketocholanic acid; (**f**) Relative abundance of indole; (**g**) Relative abundance of oleoylethanolamide; (**h**) Relative abundance of nicotinamide. *, *p* < 0.05 vs. diabetic group (Dia); **, *p* < 0.01 vs. Dia.

To explore the molecular mechanisms underlying the beneficial effects of the gut microbiota and analyze the effect of SH-P treatment on fecal metabolic profiles, untargeted metabolomics analysis by LC-MS was performed. The QC samples clustered in the center of the PCA score plots demonstrate the repeatability of the acquisition method (Figure 3a and Figure S2a). The PCA plots showed a complete distinction, and PLS-DA indicated a clear separation trend between the diabetic and SH-P-treated groups (Figure 3a,b and Figure S3a,b), indicating that the SH-P-treated group formed a distinct metabolic cluster separate from that of the diabetic group. A permutation test of the PLSDA model confirmed that the model did not overfit (Figure 3b and Figure S2b). Considering the screening criteria of variable importance in projection is >1 and a *p* < 0.05, differentially expressed metabolites were identified between the diabetic and SH-P-treated groups (Figure 3c and Figure S2c). Differential metabolites (16 and 22 in the POS and NEG modes, respectively) between the diabetic and SH-P-treated groups were visualized using heatmaps (Figure 3d and Figure S2d). Notably, SH-P treatment greatly increased metabolite levels closely associated with hyperglycemia, including those of 3-Ketocholanic acid (3.33 × 10^−4^ ± 2.27 × 10^−4^ vs. 8.19 × 10^−5^ ± 7.54 × 10^−5^, *p* < 0.05), indole (2.01 × 10^−3^ ± 3.52 × 10^−4^ vs. 9.25 × 10^−4^ ± 5.37 × 10^−4^, *p* < 0.01), oleoylethanolamide (2.37 × 10^−4^ ± 7.75 × 10^−5^ vs. 1.08 × 10^−4^ ± 6.17 × 10^−5^, *p* < 0.05), and nicotinamide (7.43 × 10^−4^ ± 3.14 × 10^−4^ vs. 2.97 × 10^−4^ ± 7.19 × 10^−5^, *p* < 0.05) in the POS mode (Figure 3e–h) and hexanoylglycine, dodecanedioic acid, 12-oxo-phytodienoic acid, 3,4-dihydroxyphenylacetic acid, and polyunsaturated fatty acids (PUFAs), such as linoleic acid and linolenic acid, in the NEG mode (Figure S2f–n).

**Figure 4:**
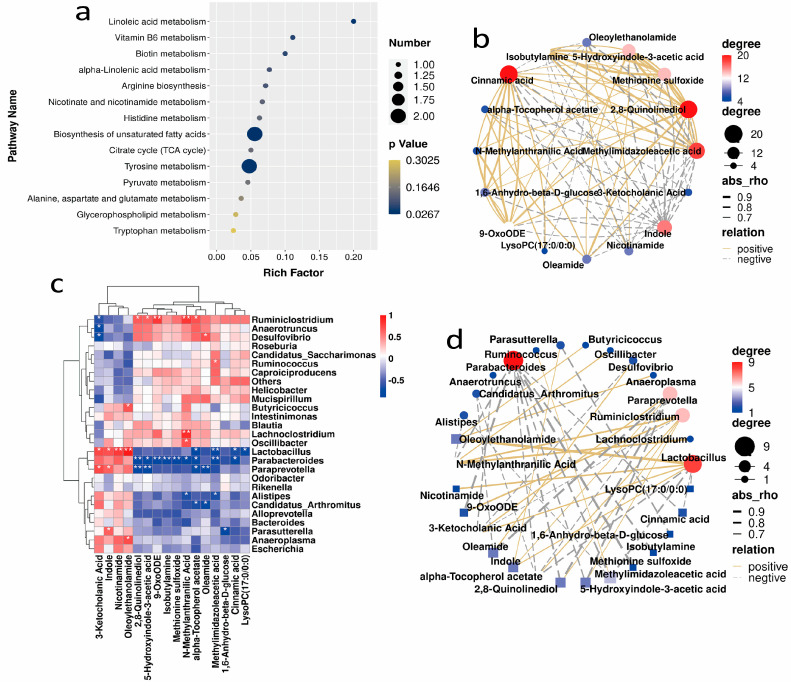
Metabolic pathways involving the differential metabolites, and Spearman’s correlation analysis of the metabolites, and the association between gut microbiota and differential metabolites. (**a**) The enriched signaling of differential metabolites between diabetic and SH-P-treated mice by KEGG enrichment analyses; (**b**) Network illustrating the interactions of the differential metabolites using the POS mode; (**c**) A hierarchical clustering heatmap showed the correlation between the dominant gut microbiota genera and differential metabolites. *, *p* < 0.05; **, *p* < 0.01; (**d**) A network illustrating the interactions between the dominant gut microbiota genera and differential metabolites.

To comprehensively investigate the metabolic pathways involved in the different metabolites, a KEGG pathway enrichment analysis of the differentially expressed metabolic pathways between the control and SH-P-treated groups was performed. This analysis indicated that SH-P treatment resulted in major alterations in metabolic pathways associated with amino acid biosynthesis and metabolic pathways, including arginine biosynthesis and histidine, tyrosine, alanine, aspartate, glutamate, and tryptophan metabolism (Figure 4a). In addition, correlations were observed between the differential metabolites 3-Ketocholanic acid, indole, oleoylethanolamide, and nicotinamide (Figure 4b), as well as 3,4-dihydroxyphenylacetic acid, 12-oxo-phytodienoic acid, hexanoylglycine, dodecanedioic acid, linoleic acid, and linolenic acid (Figure S2e).

### 2.4. Correlation Analysis between the Gut Microbiota and Metabolites in Mice

Correlations between the gut microbiota and metabolites were assessed using Spearman’s correlation analysis. Notably, in the positive detection mode, 3-Ketocholanic acid, indole, oleoylethanolamide, and nicotinamide were all positively correlated with *Lactobacillus* (Figure 4c,d). In the negative detection mode, 3,4-dihydroxyphenylacetic acid and PUFAs, such as linoleic acid and linolenic acid, which are involved in tyrosine metabolism, linoleic acid metabolism, and the biosynthesis of unsaturated fatty acids, were positively correlated with *Bacteroides*, *Lactobacillus*, and *Parabacteroides* (Figure S2o,p).

### 2.5. Effects of FMT on Alleviating Symptoms of T2DM in Mice

**Figure 5:**
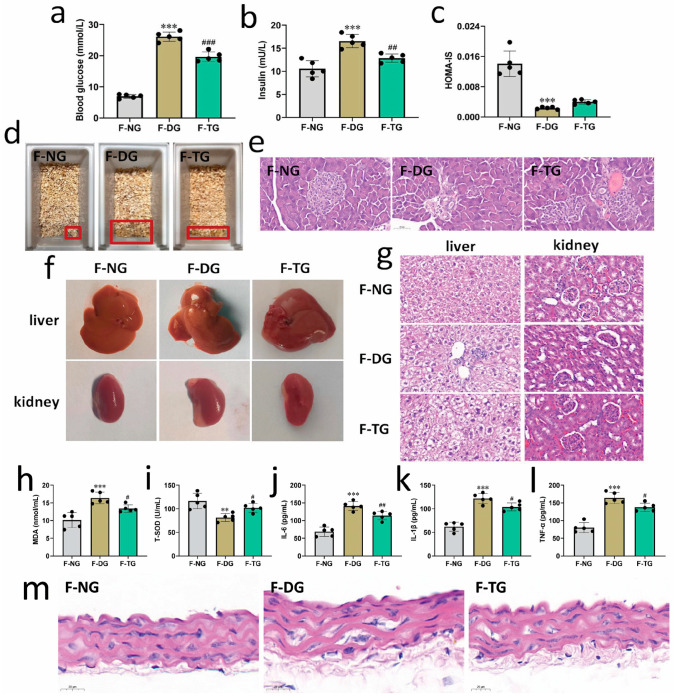
The effects of fecal microbiota transplantation (FMT) on alleviating hyperglycemia, antioxidant, and anti-inflammatory activities in diabetic mice. (**a**) Blood glucose; (**b**) Insulin; (**c**) HOMA-IS; (**d**) Urinary output; (**e**) Representative images of hematoxylin and eosin (H&E)-stained pancreas tissue; (**f**) Effects of FMT on the organ indexes of the liver and kidney; (**g**) Representative images of H&E-stained liver and kidney tissues, scale bars = 50 μm; (**h**) Malondialdehyde (MDA) content; (**i**) Total superoxide dismutase (T-SOD) activity; (**j**) IL-6 levels; (**k**) IL-1β levels; (**l**) TNF-α levels; (**m**) Representative images of the H&E-stained aorta, scale bars = 20 μm. **, *p* < 0.01 vs. normal control group (F-NG); ***, *p* < 0.001 vs. F-NG; #, *p* < 0.05 vs. diabetic control group (F-DG); ##, *p* < 0.01 vs. F-DG; ###, *p* < 0.001 vs. F-DG.

Because our findings are consistent with the supposition that SH-P treatment can improve diabetes by changing the composition of the intestinal microbiota and regulating metabolic function, we further investigated the potential effects of FMT in improving hyperglycemia. Although no significant differences in body weight and food intake were observed, the water intake of diabetic mice significantly decreased after FMT from SH-P-treated mice compared with that of the diabetic control mice (Figure S3a–c). FMT significantly reduced blood glucose and insulin levels (Figure 5a,b). Additionally, although the difference was not significant, insulin sensitivity improved in FMT mice, and the urinary output was significantly alleviated after FTM from SH-P-treated mice compared to that in the diabetic control mice (Figure 5c,d). Glucose tolerance was also ameliorated based on the OGTT and AUC in FMT mice compared with that in diabetic mice (Figure S3d,e). Moreover, FMT relieved tissue damage and dysfunction in diabetic mice. The pancreas produces insulin, which plays a major role in regulating blood sugar levels; compared to the control group, the number of islets and islet cells was significantly reduced, islet volume was significantly decreased, and the boundary was blurred in diabetic mice. FMT significantly recovered the abnormal shape and size of islets and islet cells and clearly distinguished the boundaries of the islets (Figure 5e). In addition, the liver and kidney of the diabetic control mice showed obvious swelling, and the surface of the kidney was covered with fat (Figure 5f). FMT significantly improved the swelling of the liver and kidney and reduced the fatty tissue surrounding the kidney. Histopathological analysis showed that FMT significantly improved the vacuolization and swelling of hepatocytes and alleviated glomerular volume hypertrophy and renal mesangial hyperplasia in comparison to that in the diabetic control mice (Figure 5g).

Oxidative stress and inflammation are important triggers of hyperglycemia and insulin resistance. It has been shown that polysaccharides from *P. igniarius* exhibit good anti-inflammatory and antioxidant activities [24,25]. FMT significantly decreased the MDA content (13.41 ± 1.03 vs. 16.40 ± 1.66, *p* < 0.05) and increased the T-SOD activity (102.14 ± 9.26 vs. 81.38 ± 8.30, *p* < 0.05) in the serum of FMT-treated mice compared to that in diabetic mice (Figure 5h,i). In addition, FMT also markedly reduced the levels of the pro-inflammatory cytokines IL-6 (114.05 ± 12.0 vs. 141.17 ± 12.58, *p* < 0.01), IL-1β (103.95 ± 8.55 vs. 121.99 ± 10.51, *p* < 0.05), and TNF-α (137.85 ± 11.60 vs. 164.12 ± 15.77, *p* < 0.05) compared to that in the diabetic control group (Figure 5j–l). Moreover, the arrangement of endothelial cells in the aorta was disordered, endothelial cells were swollen, the subcutaneous structure was loose, the basement membrane was discontinuous, and inflammatory cells were infiltrated in diabetic mice. However, FMT significantly alleviated aortic endothelial cell lesions and inflammatory cell infiltration, similar to that observed in normal endothelial cell structure (Figure 5m).

## 3. Discussion

Polysaccharides are basic substances that maintain the activity of living organisms and are involved in various metabolic processes. The results of this study showed that SH-P treatment significantly improved hyperglycemia symptoms and alleviated hyperglycemia-induced tissue damage. Additionally, SH-P increased insulin sensitivity and glucose tolerance in a dose-dependent manner. STZ induces selective pancreatic islet β-cell cytotoxicity and has been extensively used to induce diabetes mellitus in animals. Thus, whether the hypoglycemic mechanism of SH-P is related to regenerating the pancreatic β-cell must be further elucidated. The hypoglycemic effect of *Dendrobium huoshanense* polysaccharide was related to the improvement of pancreatic β-cell quantity and function and the regulation of hepatic glucose metabolism [26]. A polysaccharide purified from *Hovenia dulcis* was found to ameliorate type 1 diabetes mellitus (T1DM) by up-regulating PDX-1, activating and up-regulating IRS2 expression, and regulating apoptosis and regeneration of islet β-cells to recover islet β-cell function injury in T1DM rats [27]. *Tinospora cordifolia* polysaccharide possesses hypoglycemic, glucose oxidizing, and hypolipidemic abilities. In addition, it exhibits β-cell regenerative properties in the pancreatic sections [28]. A water-soluble polysaccharide obtained from pumpkin could promote the regeneration of damaged pancreatic islets by stimulating β-cell proliferation, which was accompanied by a decrease in plasma glucose levels [29]. Consequently, stimulating β-cell proliferation or inhibiting β-cell apoptosis may be one of the main reasons for the hypoglycemic effect of SH-P.

The gut microbiota is responsible for controlling energy metabolism, body weight, pro-inflammatory activity, bile acid metabolism, insulin resistance, and modulation of gut hormones [30]. In northern China, the diversity of the gut microbiota in patients with diabetes is significantly reduced compared with that in healthy individuals [31]. The decrease in gut microbiota diversity in patients with T2DM results in the malnutrition of intestinal bacteria to a certain degree, which interferes with the interaction between the gut microbiota and the host. Individuals with a lower diversity of gut microbiota composition show greater weight gain, reduced insulin sensitivity, dyslipidemia, and increased markers of inflammation [32]. Although a consensus regarding which bacteria are significantly altered is lacking, it is generally considered that the number of gut bacteria is reduced in patients with T2DM. In this study, we evaluated the effects of *P. igniarius* polysaccharides on the characteristics of gut microbiota in T2DM mice. The alpha diversity-based Chao1 and Simpson indices suggested that the *P. igniarius* polysaccharide treatment improved the species richness and evenness of the gut microbiota in diabetic mice. Moreover, PCoA based on Jaccard and Bray–Curtis distances showed that the microbial communities between the treatment and T2DM groups were clearly separated and clustered, indicating that *P. igniarius* polysaccharide treatment significantly influenced the composition of the gut microbiota. These results provide a new direction for studying the mechanisms of action of *P. igniarius* polysaccharides in the treatment of diabetes.

A significant difference in the gut microbiota between patients with T2DM and healthy individuals was observed at the phylum level [33]. The present study found that the dominant phyla in the SH-P and T2DM groups were Firmicutes and Bacteroidetes. Although the F/B ratio was not significantly different between the two groups, the proportion of Bacteroidetes in the SH-P treatment group was significantly lower than that in the T2DM group, and the proportion of Firmicutes in the T2DM group was higher than that in the SH-P treatment group. As Bacteroidetes mainly provide energy by producing acetic and propionic acids, a decrease in Bacteroidetes abundance not only reduces microbiota diversity but also disrupts the balance of energy and glucose homeostasis [34,35]. Therefore, a decrease in the abundance of Bacteroidetes may be related to the development of diabetes. At the genus level, SH-P treatment significantly increased the proportions of *Bacteroides*, *Lactobacillus*, *Alloprevotella*, *Alistipes*, and *Parabacteroides*. Interestingly, SH-P treatment also significantly increased the abundance of *Lactobacillus* from the phylum to the genus level. *Lactobacillus* is a commonly used probiotic, accounting for 6% of the total bacteria in the human duodenum and 0.3% of the total bacteria in the human colon [36,37]. *Lactobacillus* abundance appears to be related to weight gain or loss and has been shown to improve metabolic disorders caused by dietary and genetic factors, particularly impaired glucose metabolism, by enhancing intestinal barrier function and promoting the secretion of GLP-1 [38]. The development of metabolic syndrome induced by a high-fat diet is associated with decreased Arl hydrocarbon receptor (AhR) ligand levels in mice. *Lactobacillus* supplementation can efficiently activate AhR, subsequently reducing hepatic lipid accumulation and serum triglyceride levels, thereby improving lipid metabolism disorders [39]. The abundance of *Lactobacillus* is lower in adults and children with type 1 diabetes than in healthy individuals [40]. In addition to *Lactobacillus*, *Bacteroides*, *Alloprevotella*, *Alistipes*, and *Parabacteroides* are also closely related to the occurrence and development of diabetes. *Bacteroides* were considered the effective degraders for polysaccharides, which play a positive role in diabetes by up-regulating glucagon-like peptide-1 and serum insulin levels [41]. High abundances of *Alistipes* decreased the serum LDL-C, GSP, and IL-6 levels and reduced serum lipid, glucose, and inflammation marker levels, ultimately improving T2DM symptoms [42]. Hyaluronic acid increases the abundance of *Bacteroides and Alistipes* and may contribute to the decrease in fasting blood glucose [43]. *Alloprevotella* exhibits the capacity to produce short-chain fatty acids (SCFAs) and plays a positive role in alleviating inflammation [44]. Moreover, it has been elucidated that *Alloprevotella* contributes to the favorable outcomes observed in nutritional interventions targeting metabolic parameters associated with obesity [45]. *Parabacteroides* were also positively correlated with the production of SCFAs and have beneficial effects in reducing weight gain, hyperglycemia, and inflammation risk, maintaining intestinal barrier integrity, and improving insulin resistance and antioxidant enzyme activity [46]. These results indicate that SH-P treatment alleviates diabetic symptoms by improving the structure and composition of the gut microbiota and selectively restoring the abundance of probiotics while possibly also suppressing the growth of potential pathogens. However, the mechanism by which gut microbes, such as *Lactobacillus*, improve diabetes requires further study.

The gut microbiota influences human health by producing bioactive metabolites [47]. Amino acid-related metabolites, short-chain fatty acids, bile acids, trimethylamine N-oxide, and other microbial metabolites have potential effects on T2DM [11]. In this study, SH-P treatment greatly increased the levels of metabolites closely associated with hyperglycemia, including 3-Ketocholanic acid, indole, oleoylethanolamide, and nicotinamide in the POS mode, and 3,4-dihydroxyphenylacetic acid, linoleic acid, and linolenic acid in the NEG mode, demonstrating that the protective effects of SH-P treatment against diabetes are related to the regulation of microbial metabolites. KEGG pathway enrichment analysis showed that the differentially expressed metabolic pathways following SH-P treatment involved phenylalanine, tyrosine, arginine, and tryptophan biosynthesis; tryptophan, linoleic acid, nicotinate, and nicotinamide metabolism; and the biosynthesis of unsaturated fatty acids. Amino acid-derived metabolites such as these alleviate T2DM in several ways. For example, as a biosynthetic precursor of many microbial metabolites, tryptophan can be converted into indole and its derivatives by the gut microbiota. A reduction in arginine fermentation products was associated with a significant increase in fasting blood glucose and HbA1C levels in STZ-induced diabetic rats [48]. Tyrosine is metabolized to tyramine by gut microbes and is negatively correlated with inflammation biomarkers and cardiometabolic risk factors [49]. Linoleic acid, nicotinate, and nicotinamide metabolism and unsaturated fatty acid biosynthesis are all closely associated with the occurrence and development of diabetes [21,50,51,52,53]. Notably, Spearman’s correlation analysis indicated that *Lactobacillus* abundance was positively correlated with 3-Ketocholanic acid, indole, oleoylethanolamide, and nicotinamide levels; 3-Ketocholanic acid is a derivative of cholanic acid involved in the pathogenesis of T2DM and insulin resistance [54]. Indole, an interspecies signaling molecule involved in tryptophan metabolism and phenylalanine, tyrosine, and tryptophan biosynthesis, modulates the secretion of GLP-1 from intestinal enteroendocrine L-cells [55]. GLP-1 decreases blood glucose levels by stimulating insulin secretion, inhibiting glucagon secretion, and slowing gastric emptying in a glucose-dependent manner [56]. Oleoylethanolamide is an endogenous PPAR-α agonist with antihyperlipidemic, anti-inflammatory, and neuroprotective activities [33]. Nicotinamide is involved in the nicotinate and nicotinamide metabolic pathways; treatment with nicotinamide prevents or ameliorates STZ-induced diabetes and the progression of diabetes in non-obese diabetic mice [57,58]. Our results suggest that the beneficial effects of SH-P on hyperglycemia are at least partially achieved by regulating the gut microbiota, particularly the composition and metabolic functions of *Lactobacillus*. Additionally, *Bacteroides*, *Lactobacillus*, and *Parabacteroides* abundance were positively correlated with the metabolite levels of 3,4-dihydroxyphenylacetic acid, linoleic acid, and linolenic acid, which are involved in the pathways of tyrosine metabolism, linoleic acid metabolism, and the biosynthesis of unsaturated fatty acids and are all closely related to the occurrence and development of diabetes.

FMT or probiotics can improve glucose tolerance and insulin resistance by regulating gut microbiota. Clinical data have shown that probiotics can adjust the intestinal microbiota composition in a patient, which has great applicability in the treatment of diabetes [59]. In this study, FMT significantly improved hyperglycemia symptoms in T2DM mice, including decreased water intake, reduced blood glucose and insulin levels, and alleviated urinary output. Moreover, FMT ameliorated the tissue damage and dysfunction caused by diabetes. Administration of a polysaccharide isolated from *P. linteus* mycelia decreased the production of lipopolysaccharide-stimulated inflammatory cytokines, such as TNF-α, IL-1, and IL-6, in RAW264.7 mouse macrophages by regulating the PPAR-γ and mitogen-activated protein kinase (MAPK) signaling pathways [60]. *P. linteus* polysaccharides also show strong 2,2-Diphenyl-1-picrylhydrazyl free radical scavenging activity in a dose-dependent manner [61]. The activities of peroxidase dismutase, catalase, and glutathione peroxidase and MDA content in the serum and liver of senile mice significantly increased after treatment with different doses of a polysaccharide from *P. linteus* for 40 days [62]. In this study, FMT significantly decreased MDA content, increased T-SOD activity, and markedly reduced the levels of the pro-inflammatory cytokines TNF-α, IL-1β, and IL-6 in the serum of diabetic mice. Therefore, SH-P may exert its anti-inflammatory and antioxidant activities, at least in part, by regulating changes in the gut microbiota and its metabolites.

This study provides a new theoretical basis for the prevention and improvement of T2DM by regulating the gut microbiota and its metabolites, which is conducive to developing personalized interventions based on the microbiota to establish new methods for treating human metabolic diseases. However, as a special method of organ transplantation, the mechanism of action, method of use, clinical efficacy, and safety of FMT require further study. In the future, microbiota transplantation may no longer be limited to FMT but may be more focused on the specific use of different microbiota in different organs (i.e., selective microbiota transplantation), which has greater development potential in precision medicine. Additionally, the complex interplay among factors such as ethnicity, host genetics, dietary habits, and drug use plays an important role in shaping microbial communities, making it an interesting and challenging research topic.

## 4. Materials and Methods

### 4.1. Polysaccharides Isolated from P. igniarius

*P. igniarius* was dried, pulverized, and soaked in distilled water according to a solid-liquid ratio of 1:30 and reflux extracted at 90 °C for 3 h. The extraction solution was filtered using a vacuum filter pump (SHZ-DIII, Shanghai Dongxi Refrigeration Instrument Equipment Co., Ltd., Shanghai, China) and concentrated using a rotary evaporator (R205B, Shanghai SENCO Technology Co., Ltd., Shanghai, China) at 65 °C in a vacuum. The concentrated solution was mixed with anhydrous ethanol at a ratio of 1:4 (*v*/*v*) and stored overnight at 4 °C to precipitate polysaccharides. The precipitate was obtained by centrifugation at 5000× *g* for 10 min at 4 °C and dissolved in distilled water. Then, the solution was dialyzed in a dialysis bag (3500 Da mw cut-off) for 48 h at 4 °C, and the protein impurities were removed using the Sevag method. Polysaccharides (SH-P) were obtained by freeze-drying.

### 4.2. Animal Treatment

Male-specific pathogen-free grade C57/BL6J mice (20.0 ± 2.0 g) were purchased from Pengyue Experimental Animal Breeding Co., Ltd. (Jinan, China). Diabetes was induced in mice using a high-fat diet and treatment with STZ (Sigma-Aldrich, Sigma-Aldrich Co. LLC, St. Louis, MO, USA). After acclimatization for one week under a 12 h light/dark cycle at 23 ± 2 °C with a relative humidity level of 50 ± 5%, the mice were randomly divided into a normal control group (n = 5) and a diabetes group (n = 20). The control group was fed a maintenance diet, and the diabetes group was fed a high-fat diet (66.5% maintenance diet, 10% lard, 20% sucrose, 2.5% cholesterol, and 1% sodium cholate). After four weeks of feeding, the diabetes group received an intraperitoneal injection of 100 mg/kg STZ dissolved in citrate buffer (0.1 mol/L, pH 4.4) after fasting overnight. Mice with fasting blood glucose levels higher than 11.1 mmol/L after one week of STZ injection were selected as diabetic mice. Diabetic mice were further divided into five groups: a diabetes control group (n = 5), treated with 0.9% NaCl; a metformin treatment group, which received 300 mg/kg metformin; and three SH-P treatment groups orally administered SH-P at doses of 200, 400, and 800 mg/kg according to body weight once daily for four weeks. Blood glucose levels were measured weekly throughout the study. The OGTT was performed one day before euthanasia [63]. The area under the curve (AUC) and homeostasis model assessment of insulin sensitivity (HOMA-IS) were calculated using the following formulas:

$$\mathrm{AUC}\left(\frac{\min\cdot \mathrm{mmol}}{L}\right)=\left[\frac{1}{2}\times [\mathrm{BG}\left(0\;\min\right)+\mathrm{BG}\left(120\;\min\right)]\right]+\left[\mathrm{BG}\left(90\;\min\right)+\mathrm{BG}\left(60\;\min\right)-\mathrm{BG}\left(30\;\min\right)\right]\times 30\;\min$$

HOMA−IS=1/(FBG ∗ FINS)

At the end of the experiment, the mice were euthanized, and blood and tissue samples were collected for further analysis. Tissues were fixed with 4% paraformaldehyde (Biosharp, Beijing Labgic Technology Co., Ltd., Beijing, China), dehydrated in a series of graded ethanol (70, 80, 90, 95, and 100%), and embedded in paraffin. Paraffin-embedded tissues were cut into 5 μm thick slices using a microtome (RM2235, Leica Microsystems Nussloch GmbH, Nussloch, German) and stained with hematoxylin–eosin (H&E).

### 4.3. Gut Microbiota Analysis

Genomic DNA was extracted from fecal samples using DNA recovery kit (AxyPrep, Axygen Scientific Inc., San Francisco, CA, USA) according to the manufacturer’s instructions. Primers 357F (5′-ACTCCTACGGRAGGCAGCAG-3′) and 806R (5′-GGACTACHVGGGTWTCTAAT-3′) were used to amplify the V3-V4 variable region of the 16S rDNA by polymerase chain reaction, followed by purification and quantification. The amplicons were used to construct sequencing libraries, and sequencing was performed using the NGS Illumina sequencing platform (Illumina, Illumina, Inc., San Diego, CA, USA). All sequences were divided into operational taxonomic units (OTUs) with a 97% similarity cutoff using FLASH (version 1.2.11). The representative OTU sequence was compared with the database for species annotation using Mothur (classify.seqs) software (version 1.39.5). Alpha diversity was estimated using the Chao1 and Simpson indices to evaluate the species richness and diversity of the samples. Beta diversity was evaluated using principal coordinates analysis (PCoA) based on Jaccard and Bray–Curtis distances to reveal the aggregation and dispersion of samples. Linear discriminant analysis Effect Size (LEfSe) was performed to generate a cladogram for identifying different biomarkers in the gut microbiota (linear discriminant analysis (LDA) > 3, *p* < 0.05).

### 4.4. Untargeted Metabolomics Analysis by Liquid Chromatography–Mass Spectrometry

Fecal samples (50 g) were fully vortexed with 800 μL of 80% methanol. The mixtures were ground using a high-throughput tissue grinder (SCIENTZ-48, Ningbo Scientz Biotechnology Co., Ltd., Ningbo, China) at 65 Hz for 3 min and ultrasonicated (PS-80A, Shanghai Ledon Industrial Co., Ltd., Shanghai, China) in an ice-water bath at 4 °C for 30 min. Subsequently, the mixtures were stored at −40 °C for 1 h, vortexed for 30 s, centrifuged at 12,000× *g* and 4 °C for 15 min, and the supernatant was collected. Then, 200 μL of supernatant was mixed with 5 μL of dichlorophenylalanine (0.14 mg/mL, Aladdin, Shanghai Aladdin Biochemical Technology Co., Ltd., Shanghai, China) as an internal standard for further analysis. A liquid chromatography–mass spectrometry (LC-MS) platform (Waters, UPLC; Thermo Fisher Scientific, Q Exactive) and an ACQUITY HSS T3 column (2.1 × 100 mm, 1.8 μm, Waters) were used to analyze the untargeted metabolomics profiling in both ESI-positive (POS) and ESI-negative (NEG) ion modes. The gradient elution system comprised water (mobile phase A, containing 0.05% formic acid; Aladdin, China) and acetonitrile (mobile phase B; Sigma-Aldrich, USA). The column temperature was 40 °C, and the flow rate was 0.300 mL/min. The temperature of the automatic injector was 4 °C, and the injection volume was 5 μL. The mobile phase gradients are listed in Table S1. The raw data were analyzed using Compound Discoverer software (version 3.1) to obtain qualitative and quantitative results for the metabolites. Quality control (QC) was used to ensure the accuracy and reliability of the results. Principal component analysis (PCA) and partial least squares discriminant analysis (PLS-DA) were performed to evaluate the variation in metabolites in different groups. The differential metabolites were selected based on *p*-values < 0.05 (Student’s *t*-test) and the variable importance in the projection values of orthogonal projections to latent structures discriminant analysis > 1.0. Heatmaps and cluster plots were used to assess changes in the expression of the differential metabolites. Metabolic pathway enrichment of differentially expressed metabolites was determined using the Kyoto Encyclopedia of Genes and Genomes (KEGG) pathway enrichment analysis. Metabolite correlation analyses were performed to reveal the relationships between samples and metabolites. The biological significance of the metabolites was explained through a functional analysis of the metabolic pathways. Hierarchical clustering analysis was used to interpret the correlation between gut microorganisms and metabolites.

### 4.5. Fecal Microbiota Transplantation (FMT)

Fecal samples from the SH-P treatment group were collected in an empty, sterile, frozen tube using a sterile toothpick. The tube was quickly frozen in liquid nitrogen and stored at −80 °C until preparation of the fecal suspension. Fecal samples (300 mg) were dissolved in 2 mL of normal saline and homogenized by vortexing for 5 min until pellets were no longer visible. Homogenized fecal mixtures were centrifuged at 2000× *g* for 1 min at 25 °C, and the supernatants were sub-packaged into new tubes and stored at −80 °C until the day of administration. Diabetic mice were randomly divided into a diabetic control group (F-DG, n = 5) and an FMT group (F-TG, n = 5). Healthy C57/BL6J mice were selected as the normal control group (F-NG, n = 5). Mice in the FMT group were supplemented with 300 μL of the fecal suspensions by oral gavage daily for four weeks. The normal control and diabetic control mice received equivalent volumes of normal saline. Blood samples were collected to measure antioxidant parameters (malondialdehyde (MDA) and total superoxide dismutase (T-SOD) activity) and inflammatory factors (tumor necrosis factor alpha (TNF-α), interleukin-6 (IL-6), and interleukin-1β (IL-1β)) according to the manufacturer’s instructions of commercially available kits (Jiancheng, China). Tissue samples (pancreas, liver, kidney, and aorta) were collected for H&E staining.

### 4.6. Statistical Analysis

Data are expressed as the mean ± standard deviation. GraphPad Prism (version 8.0.2) was used to perform statistical analyses. One-way analysis of variance (ANOVA), followed by least significant difference analysis, was used to evaluate differences between the two groups. Differences were considered statistically significant at *p* < 0.05.

## 5. Conclusions

In conclusion, this study demonstrates that oral SH-P at a reasonable dose can alleviate hyperglycemia symptoms in mice with high-fat diet/STZ-induced diabetes. SH-P treatment significantly decreased the food and water intake, HbA1c level, and blood glucose and insulin levels and significantly improved insulin sensitivity and glucose tolerance in diabetic mice. Additionally, *P. igniarius* polysaccharide treatment decreased the F/B ratio compared to the diabetic group at the phylum level and significantly increased the proportions of *Bacteroides*, *Lactobacillus*, *Alloprevotella*, *Alistipes*, and *Parabacteroides* at the genus level. In particular, SH-P treatment significantly increased the abundance of *Lactobacillus* from the phylum to the genus level. SH-P treatment greatly increased the levels of metabolites closely associated with hyperglycemia, demonstrating that the protective effects of SH-P treatment against diabetes are related to the regulation of microbial metabolites. KEGG pathway enrichment analysis showed that the differentially expressed metabolic pathways following SH-P treatment involved phenylalanine, tyrosine, arginine, and tryptophan biosynthesis; tryptophan, linoleic acid, nicotinate, and nicotinamide metabolism; and the biosynthesis of unsaturated fatty acids. Moreover, FMT significantly relieved hyperglycemia symptoms, ameliorated tissue damage and dysfunction, and significantly improved anti-inflammatory and antioxidant activities of diabetic mice. The beneficial effects of SH-P were achieved, at least in part, by regulating the composition and metabolic functions of the gut microbiota, although future studies are required to identify the molecular and metabolic mechanisms underlying the role of SH-P in improving diabetes.

## Data Availability

The data that support the findings of this study are available from the corresponding authors upon reasonable request.

## Supplementary Materials

The following supporting information can be downloaded at: https://www.mdpi.com/article/10.3390/molecules28207136/s1, Table S1: The gradient of mobile phase; Figure S1: Gut microbiome diversity and composition analysis. (a) Relative abundance of gut microbiota at the class level; (b) Relative abundance of gut microbiota at the order level; (c) Relative abundance of gut microbiota at the family level; (d) Relative abundance of gut microbiota at the species level; Figure S2: Analysis of the differential metabolites by LC- MS between diabetic and SH-P treatment groups, the correlation of the metabolites, and the association between gut microbiota and the differential metabolites in NEG mode. (a) Score plot of PCA showing comparisons of the metabolomic profiles; (b) PLS-DA score plot of metabolomic features and validation of the PLS-DA model by permutation testing; (c) Deferentially expressed metabolites analysis by volcano plot. The up-regulated metabolites are indicated in red, the down-regulated ones are in blue, and the gray dots denote the insignificant metabolites; (d) Hierarchical clustering heatmap exhibiting the metabolites that differ significantly in abundance; (e) Network illustrating the interactions of the differential metabolites; (f) Relative abundance of linoleic acid; (g) Relative abundance of 5-hydroxyindole-3-acetic acid; (h) Relative abundance of gamma-glutamylleucine; (i) Relative abundance of dodecanedioic acid; (j) Relative abundance of 3-methylglutaric acid; (k) Relative abundance of 3,4-dihydroxyphenylacetic acid; (l) Relative abundance of 12-oxo phytodienoic acid; (m) Relative abundance of linolenic acid; (n) Relative abundance of hexanoylglycine. *, *p* < 0.05, vs. Dia; **, *p* < 0.01, vs. Dia. (o) Hierarchical clustering heatmap showed the correlation between the dominant gut microbiota genera and differential metabolites; (p) Network illustrating the interactions between the dominant gut microbiota genera and differential metabolites. Significant correlations are indicated as * *p* < 0.05 and ** *p* < 0.01; Figure S3: Effect of FMT on relieving hyperglycemic symptoms. (a) Body weight; (b) Food intake; (c) Water intake; (d) OGTT; (e) AUC. **, *p* < 0.01, vs. F-NG; ***, *p* < 0.001, vs. F-NG; #, *p* < 0.05, vs. F-DG; ##, *p* < 0.01, vs. F-DG.
