# Supplementary material for: Phellinus igniarius Polysaccharides Ameliorate Hyperglycemia by Modulating the Composition of the Gut Microbiota and Their Metabolites in Diabetic Mice
Source: Molecules. 2023 Oct 17;28(20):7136. doi: 10.3390/molecules28207136 (PMC10609020; doi:10.3390/molecules28207136)
Supplement: Supplementary file 1 [file molecules-28-07136-s001.zip › molecules-2634099-supplementary.pdf]

Table S1 The gradient of mobile phase

Time (min)	Flow rate (mL/min)	A (%)	B (%)
0.00	0.30	95	5
1.00	0.30	95	5
12.50	0.30	5	95
13.50	0.30	5	95
13.60	0.30	95	5
16.00	0.30	95	5

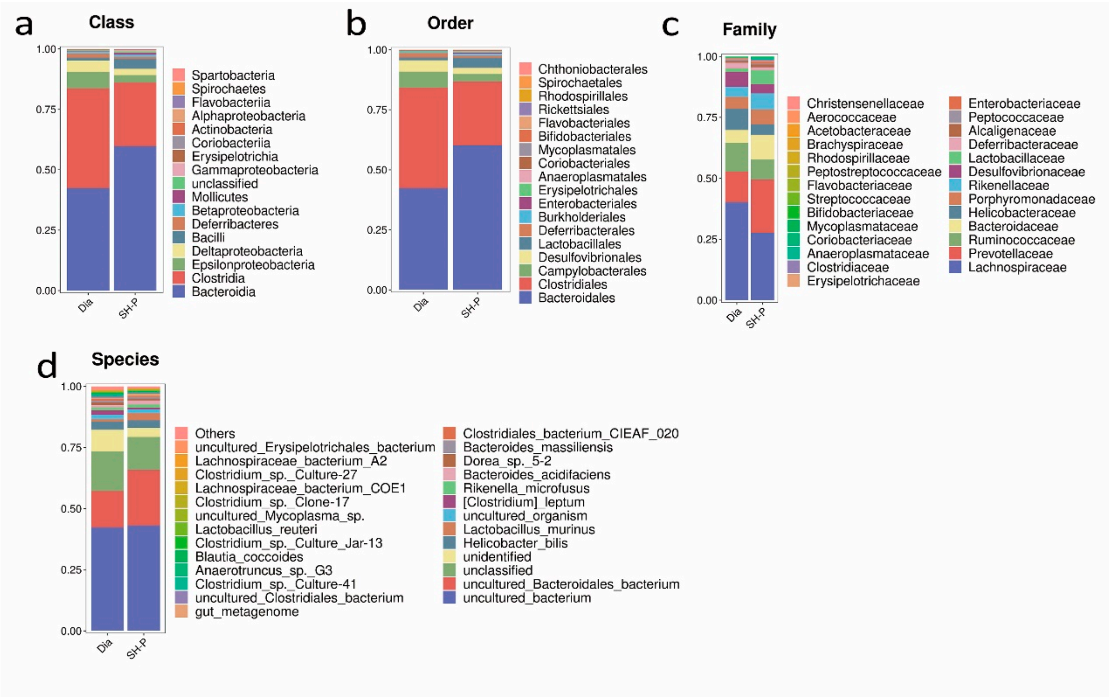

Figure S1. Gut microbiome diversity and composition analysis. (a) Relative abundance of gut microbiota at the class level; (b) Relative abundance of gut microbiota at the order level; (c) Relative abundance of gut microbiota at the family level; (d) Relative abundance of gut microbiota at the species level.

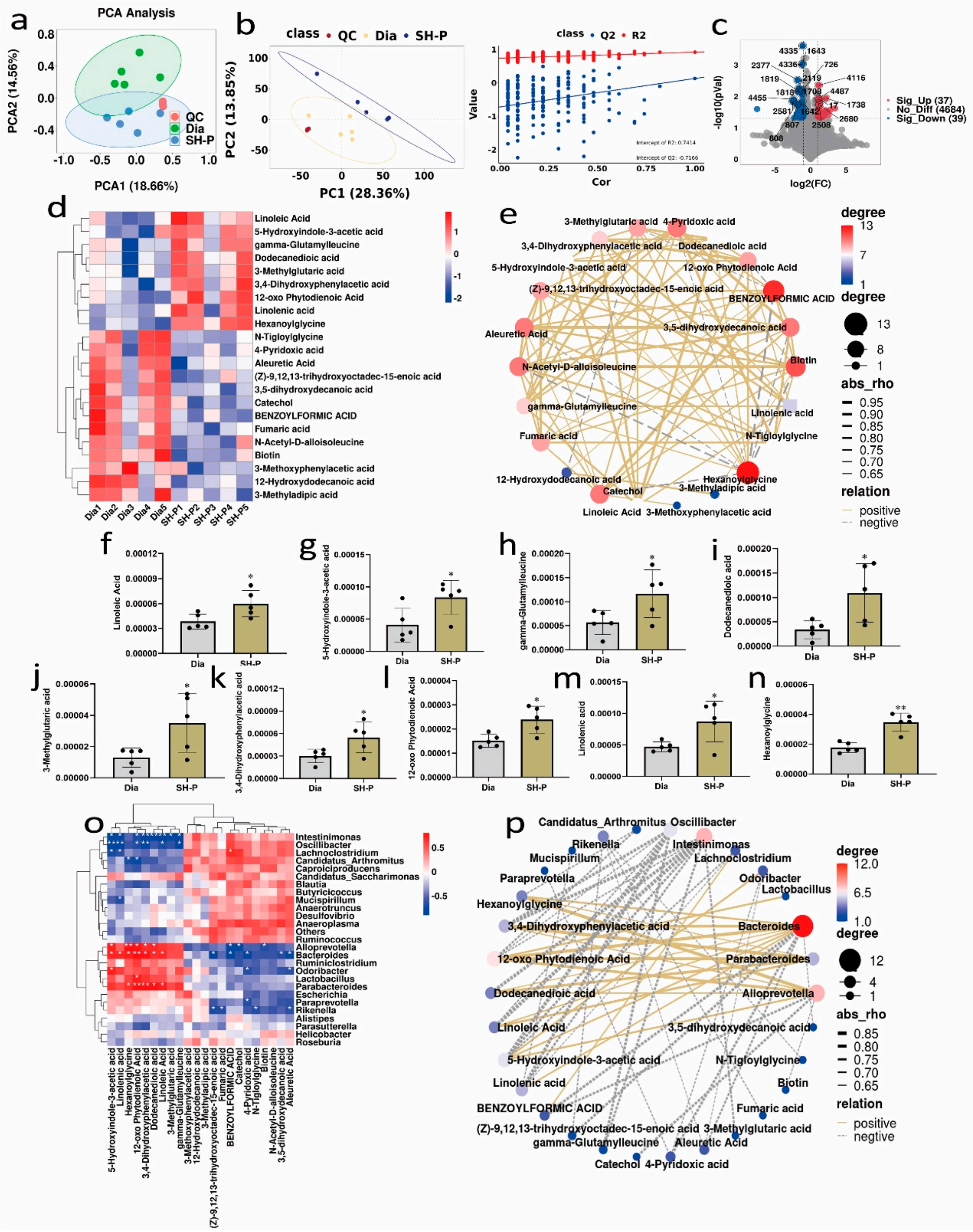

Figure S2. Analysis of the differential metabolites by LC- MS between diabetic and SH-P treatment groups, the correlation of the metabolites, and the association between gut microbiota and the differential metabolites in NEG mode. (a) Score plot of PCA showing comparisons of the metabolomic profiles; (b) PLS-DA score plot of metabolomic features and validation of the PLS-DA model by permutation testing; (c) Deferentially expressed metabolites analysis by volcano plot. The up-regulated metabolites are indicated in red, the down-regulated ones are in blue, and the gray dots denote the insignificant metabolites; (d) Hierarchical clustering heatmap exhibiting the metabolites with significantly differ in abundance; (e) Network illustrating the interactions of the differential metabolites; (f) Relative abundance of linoleic acid; (g) Relative abundance of

5-hydroxyindole-3-acetic acid; (h) Relative abundance of gamma-glutamylleucine; (i) Relative abundance of dodecanedioic acid; (j) Relative abundance of 3-methylglutaric acid; (k) Relative abundance of 3,4-dihydroxyphenylacetic acid; (l) Relative abundance of 12-oxo phytodienoic acid; (m) Relative abundance of linolenic acid; (n) Relative abundance of hexanoylglycine. *, p<0.05, vs. Dia; **, p<0.01, vs. Dia. (o) Hierarchical clustering heatmap showed the correlation between the dominant gut microbiota genera and differential metabolites; (p) Network illustrated the interactions between the dominant gut microbiota genera and differential metabolites. Significant correlations are indicated as * p < 0.05 and ** p < 0.01.

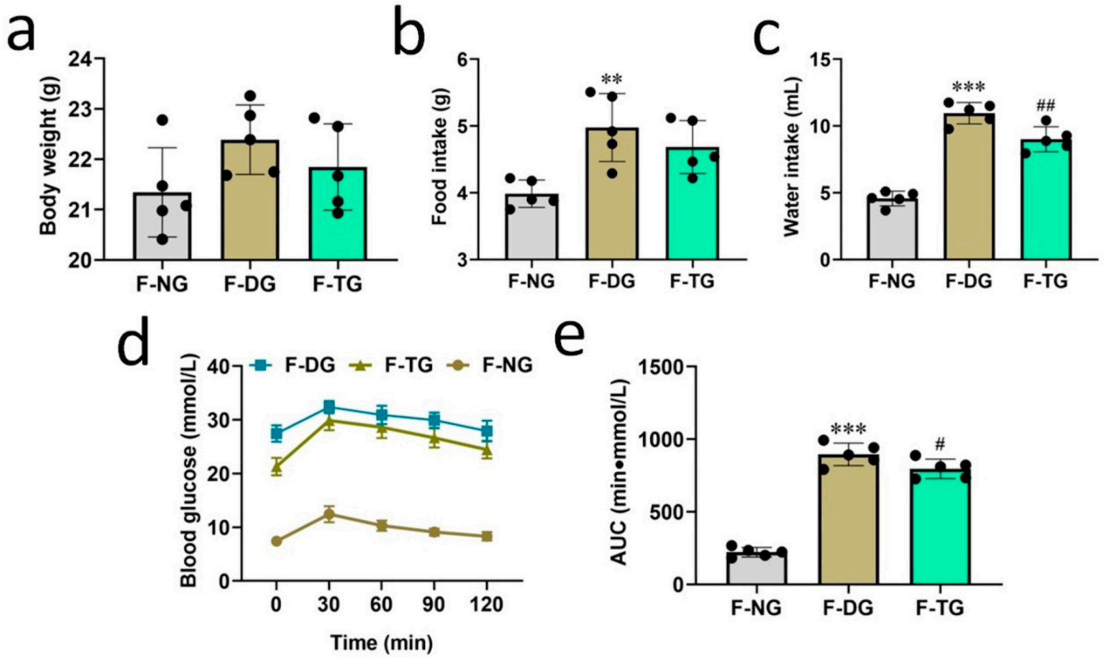

Figure S3. Effect of FMT on relieving hyperglycemic symptoms. (a) Body weight; (b) Food intake; (c) Water intake; (d) OGTT; (e) AUC. **, p<0.01, vs. F-NG; ***, p<0.001, vs. F-NG; #, p<0.05, vs. F-DG; ##, p<0.01, vs. F-DG.
